# Ado-Trastuzumab Emtansine in Metastatic HER2-Positive Breast Cancer

**DOI:** 10.6004/jadpro.2014.5.2.5

**Published:** 2014-03-01

**Authors:** Rena Callahan

**Affiliations:** From UCLA Medical Center, Hematology Oncology, Los Angeles, California

Breast cancer is the most common cancer in US women, affecting approximately 1 in 8 women over the course of a lifetime. Nearly 40,000 women die of breast cancer every year (Siegel, Naishadham, & Jemal, 2013). In the past decade, we have come to understand that breast cancer is a heterogeneous disease, comprising subtypes that are defined by gene expression profiling (Perou et al., 2000). Treatment choices such as chemotherapy, endocrine therapy, and therapies targeted toward specific genetic alterations have resulted in improved survival.

Approximately 25% of all breast cancers are classified as HER2 positive, in which there is overexpression of the HER2 protein (as measured by immunohistochemistry) and/or amplification of the HER2 gene (determined by fluorescence in situ hybridization). HER2 activation triggers an intracellular signaling cascade, which results in cell proliferation, survival, invasion, and angiogenesis. HER2-positive breast cancers have an aggressive phenotype. However, with the advent of HER2-targeted therapy, the natural history of HER2-positive breast cancer has been dramatically improved (Ferretti, Fabi, Felici, & Papaldo, 2010; Dawood, Broglio, Buzdar, Hortobagyi, & Giordano, 2010).

## HER2-Directed Therapies

Trastuzumab (Herceptin) is a monoclonal antibody that targets the HER2 receptor tyrosine kinase. In a study of 469 patients with meta-static breast cancer randomized to receive standard chemotherapy vs. chemotherapy plus trastuzumab, the group receiving trastuzumab demonstrated response rates of 50% vs. 32% and median survival times of 25.1 vs. 20.3 months, respectively (Slamon et al., 2001). Trastuzumab was approved by the US Food and Drug Administration (FDA) for use in patients with metastatic breast cancer in 1998. It was subsequently evaluated in the nonmetastatic setting and showed striking benefits in relapse-free survival and overall survival (OS), leading to its approved use in patients with early-stage HER2-positive breast cancer in 2006 (Romond et al., 2005; Piccart-Gebhart, 2005).

Despite the benefits of adjuvant trastuzumab for HER2-positive breast cancer, recurrences do occur. For these patients, resistance to trastuzumab ultimately develops, and they eventually succumb to their disease. Therefore, there was a need to develop other drugs for patients who develop trastuzumab resistance.

Lapatinib (Tykerb) was the second HER2-directed agent approved by the FDA for use in metastatic breast cancer. It is a small-molecule tyrosine kinase inhibitor that targets the intracellular portion of the HER2 receptor. Its approval was largely based on a study of patients with advanced breast cancer whose disease had progressed on an anthracycline, a taxane, and trastuzumab; patients were randomized to receive either capecitabine or capecitabine with lapatinib. The addition of lapatinib improved median time to progression from 4.4 to 8.4 months, and the combination was approved by the FDA in March 2007 (Geyer et al., 2006). Since that time, studies evaluating the use of trastuzumab with varying chemotherapeutic agents have been performed, and it has been incorporated into widespread use (Slamon et al., 2011). The use of lapatinib in combination with endocrine therapy has also been evaluated and is approved for use in metastatic HER2-positive breast cancer.

In June 2012, pertuzumab (Perjeta), a new monoclonal antibody that binds and blocks the action of the HER2 receptor in a manner complementary to that of trastuzumab, was approved for use in first-line metastatic HER2- positive breast cancer in combination with taxane chemotherapy and trastuzumab. This approval was based on a demonstrable benefit in progression-free survival (PFS; 18.5 vs. 12.4 months) when pertuzumab was added to docetaxel and trastuzumab (Baselga et al., 2012).

The newest agent in the armamentarium against HER2-positive breast cancer is ado-trastuzumab emtansine (Kadcyla), also known as T-DM1, which earned FDA approval in February 2013 for metastatic HER2-positive breast cancer that has progressed on a trastuzumab-containing regimen (Genentech, 2013). See the Table on the next page for a summary of key information.

**Table 1:**
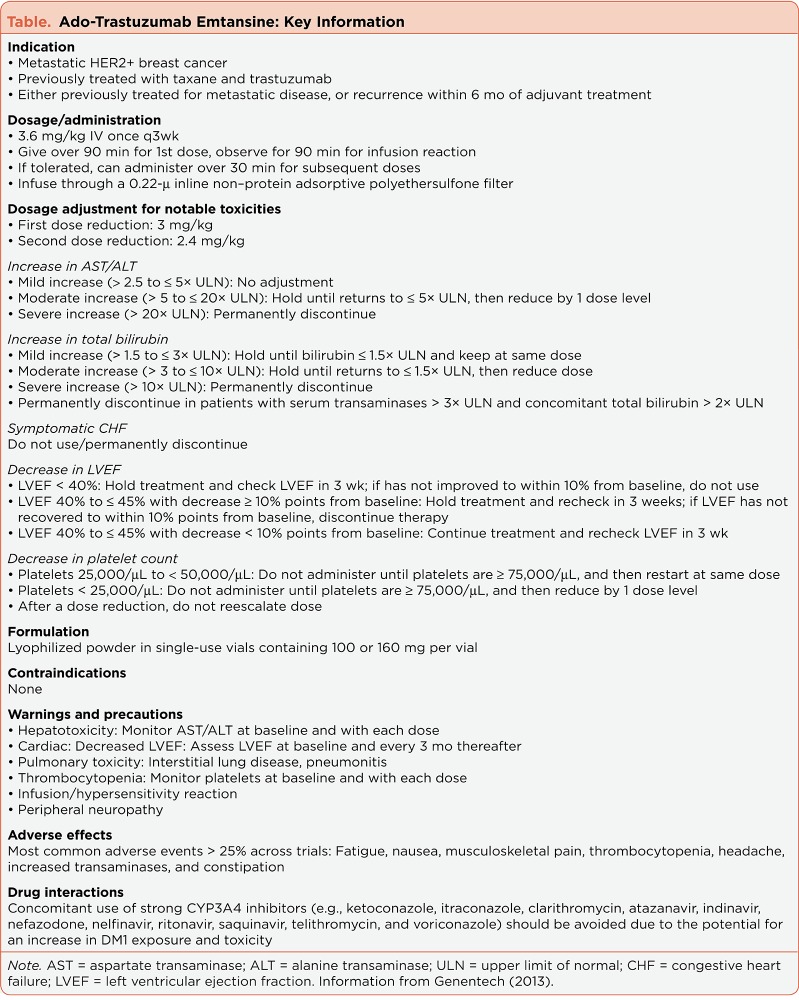
Table. Ado-Trastuzumab Emtansine: Key Information

## Mechanism of Action

Ado-trastuzumab emtansine is described as an antibody drug conjugate (ADC). It contains trastuzumab attached to a chemotherapy molecule (DM1) by a stable linker molecule. Trastuzumab is a humanized, anti-HER2 IgG1 antibody. The DM1 chemotherapy molecule is a maytansine derivative that was studied in the 1970s at the National Cancer Institute. Development was halted at that time due to unacceptable toxicity in human trials. However, in ado- trastuzumab emtansine, a thioether linker (MCC) stabilizes the compound so that the DM1 chemotherapy molecule is targeted to the HER2+ cancer cell. The chemotherapy is internalized and released into that cell only after it is attached to the HER2+ cell. Therefore, the cancer cells are destroyed, and surrounding normal tissue is spared the toxicity of the chemotherapy.

Ado-trastuzumab emtansine demonstrates mechanisms of action of both trastuzumab and DM1. In vitro, similar to trastuzumab, it inhibits HER2 receptor signaling and enhances antibody-dependent cell-mediated cytotoxicity. Ado- trastuzumab emtansine, like trastuzumab, inhibits shedding of the HER2 extracellular domain, which is a known mechanism of resistance to trastuzumab. The DM1 chemotherapy molecule, upon internalization and release into the cancer cell, inhibits microtubule function, thereby interfering with progression through the cell cycle (Baselga et al., 2012).

## Clinical Studies

A phase I, multicenter, open-label, dose-escalation study of single-agent ado-trastuzumab emtansine was initiated to assess its safety, tolerability, and pharmacokinetics in patients with HER2-positive metastatic breast cancer who had previously received trastuzumab (Krop et al., 2010).

Twenty-four patients were treated at doses ranging from 0.3 to 4.8 mg/kg. In this study, 3.6 mg/kg was identified as the maximum tolerated dose (MTD). The dose-limiting toxicity was thrombocytopenia, yet it was usually mild (grade 1 or 2) and rapidly reversible upon discontinuation of therapy. Platelet count usually dropped starting 1 day after ado- trastuzumab emtansine administration, with a nadir around day 8 and recovery by day 15. There were no bleeding events that required transfusion or any other intervention.

Overall, the most commonly reported adverse events (AEs) were thrombocytopenia (54.2%), elevated transaminases (41.7%), fatigue (37.5%), anemia (29.2%), and nausea (25.0%). No patients experienced grade > 1 nausea, vomiting, alopecia, or neuropathy. The overall response rate (ORR) was 44% in the 9 patients with measurable disease treated at the MTD. The clinical benefit rate (CBR) was 73% (ORR + stable disease) among the 15 patients treated at the MTD. The median duration of treatment was 238 days for these 15 patients.

Based on the favorable phase I safety and efficacy profile, the clinical development of ado-trastuzumab emtansine continued. A phase II, open-label, single-arm study of ado-trastuzumab emtansine (TDM4374g) included 110 patients who had previously been treated with trastuzumab, lapatinib, an anthracycline, a taxane, and capecitabine. The primary objectives were ORR and safety. On average, patients had been treated for metastatic breast cancer with seven prior agents. The ORR was 34.5% and the CBR was 48.2%. The median PFS was 6.9 months in this heavily pretreated group of patients (Krop et al., 2012). In another phase II, open-label, single-arm study of ado-trastuzumab emtansine (TDM4258g) that included 112 patients, ORR was 26% and median PFS was 4.6 months (Burris et al., 2011).

The EMILIA study was a phase III, randomized, multicenter, open-label trial of ado-trastuzumab emtansine vs. capecitabine plus lapatinib in 991 women with metastatic HER2-positive breast cancer who progressed on or within 6 months of having received trastuzumab and who had received taxane chemotherapy (Verma et al., 2012). Patients were randomly allocated (1:1) to receive ado-trastuzumab emtansine 3.6 mg/kg once every 21 days or oral lapatinib 1,250 mg/day once daily plus oral capecitabine 1,000 mg/m^2^ twice daily for 14 of every 21 days. The primary endpoints were PFS and OS. A total of 88% of the patients had received prior systemic treatment in the metastatic setting; 12% of the patients had prior treatment only in the neoadjuvant or adjuvant setting with disease relapse within 6 months. Patients received a median of three systemic agents for metastatic disease.

Median PFS was 9.6 months in the ado-trastuzumab emtansine group vs. 6.4 months in the lapatinib plus capecitabine group. The hazard ratio for PFS was 0.65 with ado-trastuzumab emtansine vs. capecitabine plus lapatinib. Median duration of survival was 30.9 months in the ado-trastuzumab emtansine group vs. 25.1 months in the lapatinib plus capecitabine group (Verma et al., 2010).

More recently, results from TDM4450g, a phase II, multicenter, open-label study of ado-trastuzumab emtansine in the first-line setting, were published. This was the first trial to directly compare ado-trastuzumab emtansine with a standard trastuzumab plus chemotherapy regimen in patients who had not received treatment for metastatic disease. In this trial, 137 patients with advanced HER2-positive breast cancer were randomized in a 1:1 ratio to receive ado- trastuzumab emtansine 3.6 mg/kg IV once every 3 weeks vs. trastuzumab 8 mg/kg loading dose then 6 mg/kg IV and docetaxel 75 or 100 mg/m^2^ (HT) at the investigator’s discretion, once every 3 weeks. The primary endpoint was PFS and safety. Secondary endpoints included OS, ORR, duration of response (DOR), CBR, and quality of life (QOL). Of note, only 27.1% and 17.9% of patients in the ado-trastuzumab emtansine and HT groups, respectively, had received prior trastuzumab in the adjuvant or neoadjuvant setting. The ado-trastuzumab emtansine group demonstrated a median PFS of 14.2 months compared with 9.2 months in the HT group. The hazard ratio for PFS was 0.59 with ado- trastuzumab emtansine vs. HT. The ORR and CBR in the ado-trastuzumab emtansine group was 64.2% vs. 58% in the HT group (Hurvitz et al., 2013).

## Adverse Events

In the single-arm, phase II study TDM4374g, fatigue (61.8%), nausea (37.3%), and thrombocytopenia (38.2%) were among the most common adverse events (AEs) seen. Thrombocytopenia (9.1%), fatigue (4.5%), and cellulitis (3.6%) were the most common grade 3 adverse events. In TDM4258g, the other single-arm phase II study, the most frequent grade 3 AEs were hypokalemia (8.9%), thrombocytopenia (8.0%), and fatigue (4.5%; Krop et al., 2012).

In the EMILIA study, grade 3 adverse events were more common with capecitabine plus lapatinib vs. ado- trastuzumab emtansine (57% vs. 40.8%). The most commonly reported grade 3 AEs in the capecitabine plus lapatinib group were diarrhea (20.7%) and palmar-plantar erythrodysesthesia (16.4%). Thrombocytopenia and elevated serum concentrations of aspartate transaminase (AST) and alanine transaminase (ALT) were the most commonly reported grade 3 adverse events in the ado-trastuzumab emtansine group, affecting 12.9%, 4.3%, and 2.9% of patients, respectively.

While thrombocytopenia is a common side effect of ado-trastuzumab emtansine, it led to discontinuation of therapy in only 2% of patients in the EMILIA study. Thrombocytopenia was usually seen in the first two cycles of therapy, responded to dose reductions, and was rarely associated with significant bleeding. The rate of grade 3 or 4 bleeding events was low overall in the ado-trastuzumab emtansine group (1.4%, vs. 0.8% in the capecitabine plus lapatinib group).

In the phase II TDM4450g trial, treatment with ado-trastuzumab emtansine also resulted in fewer grade ≥ 3 adverse events than with the comparator—in that case, HT (AEs: 46.4% vs. 90.9%). Grade 4 AEs occurred in 57.6% of patients receiving HT vs. 5.8% in patients receiving ado-trastuzumab emtansine. A total of 40.9% of patients in the HT group experienced AEs leading to treatment discontinuation (of either drug) vs. 7.2% in the ado-trastuzumab emtansine group. In the HT group, the most common AEs of any grade were alopecia, neutropenia, diarrhea, and fatigue. The most common AEs in the ado-trastuzumab emtansine group were fatigue, nausea, increase in serum AST, fever, and headache. A notable difference in side-effect profile between ado-trastuzumab emtansine and taxane-based chemotherapy with trastuzumab is the lack of alopecia seen with ado-trastuzumab emtansine. Consistent with the AE profile of both study arms, QOL assessments in this study favored ado-trastuzumab emtansine over HT (Hurvitz et al., 2013).

## Cardiotoxicity

Studies of therapy with ado-trastuzumab emtansine took special note of cardiac events due to the known potential for trastuzumab-associated cardiotoxicity. With trastuzumab, this cardiotoxicity, manifest by decreases in left ventricular ejection fraction (LVEF), is usually asymptomatic and readily reversible. Additionally, trastuzumab-associated cardiotoxicity is worsened in patients receiving concurrent or prior anthracycline therapy (Romond et al., 2012).

In a single-arm phase II study (TDM4374g), at a median follow-up of 17.4 months, no left ventricular ejection fraction decline to ≤ 45% or symptomatic congestive heart failure was observed, and no patients discontinued treatment because of cardiotoxicity. Similarly, in TDM4258g, there was no dose-limiting cardiotoxicity observed (Burris et al., 2011). In the randomized TDM4450g trial, there were no clinically significant cardiac events in either group. At a median follow-up of 23 months, 3 patients—1 in the ado-trastuzumab emtansine group and 2 in the HT group—had an asymptomatic decline in LVEF to ≤ 40% (Hurvitz et al., 2013).

Primarily because it contains trastuzumab, the FDA-approved prescribing information recommends that all patients treated with ado-trastuzumab emtansine have a baseline LVEF assessment at baseline and at regular intervals (e.g., every 3 months) during treatment (Genentech, 2013). At least temporary discontinuation of therapy is recommended if the LVEF falls to < 40% or is 40% to 45% with a 10% absolute decrease below the pretreatment value. In this case, LVEF should be rechecked 3 weeks later. If it has not improved or worsened, then permanent discontinuation is recommended. However, if there is improvement, it is acceptable to restart ado-trastuzumab emtansine with close cardiac monitoring.

## Summary

In a phase III study of patients with HER2-positive metastatic breast cancer previously treated with trastuzumab, ado-trastuzumab emtansine resulted in a 35% reduction in the risk of progressive disease compared with standard treatment (Verma et al., 2012). In the phase II trial of ado-trastuzumab emtansine used in the first-line setting, there was a 41% reduction in risk of progressive disease compared with a standard therapy (Hurvitz et al., 2013). Remarkably, these improvements in efficacy came with a decrease in toxicity vs. the comparator arm.

Ado-trastuzumab emtansine is an effective, tolerable, IV, "smart" therapy, now approved for use in patients with HER2-positive breast cancer who have progressed on or within 6 months of a trastuzumab-containing treatment regimen. Ongoing studies are evaluating its use as first-line therapy for metastatic disease, in combination with other HER2-directed therapies and in the neoadjuvant setting. The use of ado-trastuzumab emtansine may be expanded if these trials demonstrate safety and efficacy in these studies. Therefore, the use of ado-trastuzumab emtansine is expected to increase dramatically. In light of this, it is essential for the oncology advanced practitioner to understand its mechanism of action, efficacy, and adverse event profile.
